# ABAS1 from soybean is a 1R-subtype MYB transcriptional repressor that enhances ABA sensitivity

**DOI:** 10.1093/jxb/eraa081

**Published:** 2020-02-15

**Authors:** Yee-Shan Ku, Meng Ni, Nacira B Muñoz, Zhixia Xiao, Annie Wing-Yi Lo, Pei Chen, Man-Wah Li, Ming-Yan Cheung, Min Xie, Hon-Ming Lam

**Affiliations:** 1 Centre for Soybean Research of the State Key Laboratory of Agrobiotechnology and School of Life Sciences, The Chinese University of Hong Kong, Hong Kong Special Administrative Region, China; 2 Instituto de Fisiología y Recursos Genéticos Vegetales, Centro de Investigaciones Agropecuarias–INTA, Córdoba, Argentina; 3 Cátedra de Fisiología Vegetal, Facultad de Ciencias Exactas Físicas y Naturales, Universidad Nacional de Córdoba, Córdoba, Argentina; 4 Agro-biological Gene Research Center, Guangdong Academy of Agricultural Sciences, Guangzhou, China; 5 University of Essex, UK

**Keywords:** ABA, abiotic stress, MYB, soybean, stomatal aperture, transcription factor

## Abstract

Transcription factors (TFs) help plants respond to environmental stresses by regulating gene expression. Up till now, studies on the MYB family of TFs have mainly focused on the highly abundant R2R3-subtype. While the less well-known 1R-subtype has been generally shown to enhance abscisic acid (ABA) sensitivity by acting as transcriptional activators, the mechanisms of their functions are unclear. Here we identified an ABA sensitivity-associated gene from soybean, *ABA-Sensitive 1* (*GmABAS1*), of the 1R-subtype of MYB. Using the GFP-GmABAS1 fusion protein, we demonstrated that GmABAS1 is localized in the nucleus, and with yeast reporter systems, we showed that it is a transcriptional repressor. We then identified the target gene of GmABAS1 to be *Glyma.01G060300*, an annotated *ABI five-binding protein 3* and showed that GmABAS1 binds to the promoter of *Glyma.01G060300* both *in vitro* and *in vivo*. Furthermore, *Glyma.01G060300* and *GmABAS1* exhibited reciprocal expression patterns under osmotic stress, inferring that GmABAS1 is a transcriptional repressor of *Glyma.01G060300*. As a further confirmation, *AtAFP2*, an orthologue of *Glyma.01G060300*, was down-regulated in *GmABAS1*-transgenic *Arabidopsis thaliana*, enhancing the plant’s sensitivity to ABA. This is the first time a 1R-subtype of MYB from soybean has been reported to enhance ABA sensitivity by acting as a transcriptional repressor.

## Teaser

ABAS1 negatively regulates the expression of a soybean *AFP3* homologue and enhances ABA sensitivity. This is the first report on the transcriptional repressor activity of a 1R-subtype MYB from soybean.

## Introduction

In response to environmental challenges, transcription factors play crucial roles in regulating gene expressions (Singh *et al.*, 2002; Golldack *et al.*, 2011). Among different classes of transcription factors, myeloblastosis (MYB), a subtype of homeodomain-like proteins, comprises a large group of transcription factors found in plants including soybean (Chai *et al.*, 2015). According to the number and type of the helix–turn–helix (HTH) motifs, MYB-type transcription factors are further divided into various subtypes, including 1R, R2R3, 3R, and 4R (Dubos *et al.*, 2010; Du *et al.*, 2012). Most plant studies have been focused on the R2R3 subtype due to its high abundance in the genome (Yanhui *et al.*, 2006; Du *et al.*, 2012). 1R-subtype MYBs typically contain one HTH domain near the N-terminus (Ambawat *et al.*, 2013) and a regulatory region at the C-terminus modulating transcriptional activities (Du *et al.*, 2009).

Compared with other types of MYBs, there are only limited functional studies on the 1R-subtype MYBs in plants. The first plant 1R-subtype MYB, MybSt1, was identified as a transcriptional activator from potato (Baranowskij *et al.*, 1994). When overexpressed, the potato *StMYB1R-1* gene could enhance stomatal closure under abscisic acid (ABA) treatment (Shin *et al.*, 2011). In tomato, a mutation in the *ars1* gene (encoding a 1R-subtype MYB) led to reduced sensitivity toward ABA (Campos *et al.*, 2016). The rice *OsMYB48-1* gene, which encodes a single SANT (Swi3, Ada2, N-Cor, and TFIIIB) domain, is a 1R-subtype MYB protein and a transcriptional activator that enhances ABA sensitivity (Xiong *et al.*, 2014). These findings suggest the role of 1R-subtype MYB proteins in regulating ABA sensitivity. However, in most of these previous reports, the transcriptional regulatory activities of the 1R-subtype MYBs or their putative transcriptional regulatory targets remained untested.

In soybean, a few 1R-subtype MYBs have been identified (Dhaubhadel and Li, 2010; Yi *et al.*, 2010; Li and Dhaubhadel, 2012; Bian *et al.*, 2017). GmMYB176 was proposed to be a transcriptional activator, while the transcriptional activities of GmMYB62 and GmMYB138a were not reported. Despite the fact that a genome-wide survey of MYB-type transcription factors in soybean has been performed, no new members of the 1R-subtype MYB were found (Du *et al.*, 2012). The putative functions of 1R-subtype MYBs in soybean remain underexplored.

Our previous genomic studies on a wild soybean×cultivated soybean recombinant inbred population have led to the identification of a salt tolerance quantitative trait locus (QTL) (Qi *et al.*, 2014). In a survey of genes close to the salt tolerance QTL on soybean chromosome 3, we identified a 1R-subtype MYB, *ABA-Sensitive1* (*GmABAS1*). Interestingly, *GmABAS1* encodes a full-length protein in the wild soybean parent while having a 19 bp deletion in the cultivated parent. The gene having the 19 bp deletion was named *GmABAS1∆*. Here, we report that GmABAS1 exhibits transcriptional repressor activity and regulates ABA sensitivity.

## Materials and methods

### RNA extraction, cDNA synthesis, genomic DNA extraction, cloning, and sequence analysis

Total RNA was isolated from soybean or *Arabidopsis thaliana* tissues by TRIzol™ Reagent (15596026, ThermoFisher Scientific) according to the manufacturer’s protocol. The total RNA was then treated with DNase I (18068-015, ThermoFisher Scientific) according to the manufacturer’s protocol. To amplify *GmABAS1* and *GmABAS1∆* from soybean germplasms for sequence comparisons and expression analyses, total cDNAs were synthesized using PrimeScript™ RT Master Mix (Perfect Real Time) (RR036Q, TaKaRa) from the total RNA according to the manufacturer’s protocol. To clone *GmABAS1*, *GmABAS1∆*, *AtVIP1*, and *AtDET1*, total cDNAs were synthesized using the SuperScript™ III Reverse Transcriptase (18080093, ThermoFisher Scientific) with oligo(dT)_20_ according to the manufacturer’s protocol.

A 3 kb putative promoter sequence upstream of the translational start of *Glyma.01G060300* was amplified from the genomic DNA of the wild soybean germplasm, W05. Genomic DNA was extracted from the leaves of W05 plants using the cetyltrimethylammonium bromide (CTAB) method (Doyle and Doyle, 1987). PCR was performed using PrimeSTAR GXL DNA Polymerase (R050B, TaKaRa).

The cDNAs and genomic promoter sequence were cloned into various vectors for *Escherichia coli* or yeast transformation. Restriction enzymes and T4 DNA ligase used in cloning were from New England Biolabs.

For DNA sequence analyses, *GmABAS1* and *GmABAS1∆* were amplified from total cDNAs using iProof™ High-Fidelity DNA Polymerase (1725301, Bio-Rad). The PCR products were subjected to standard sequencing via a commercial service (Macrogen, Seoul). MAFFT (https://www.ebi.ac.uk/Tools/msa/mafft/) was used for DNA sequence alignment. The ExPASy translation tool (https://web.expasy.org/translate/) was used for *in silico* translation. MEGA7 was used for protein sequence alignments and phylogenetic analyses. For protein domain analyses, ExPASy PROSITE (https://prosite.expasy.org/) and cNLS Mapper (http://nls-mapper.iab.keio.ac.jp/cgi-bin/NLS_Mapper_form.cgi) were used. For protein structure analyses, Phyre2 (Kelley *et al.*, 2015) was used.

Primers and vectors used are listed in Supplementary Tables S1 and S2 at *JXB* online.

### Yeast transformations and western blot analyses

Yeast cells were transformed using the LiAc method according to the Yeast Protocols Handbook (TaKaRa). Total yeast proteins were extracted using the urea/SDS method according to the Yeast Protocols Handbook (TaKaRa) in a buffer containing Halt™ Protease Inhibitor Cocktail, EDTA-Free (87785, ThermoFisher Scientific). Total yeast proteins were electrophoresed by SDS–PAGE on the TGX Stain-Free™ FastCast™ acrylamide gel (12%, 1610175, Bio-Rad), and then visualized using the Gel Doc™ EZ Gel Documentation System (Bio-Rad). Precision Plus Protein™ Unstained Standards (1610394, Bio-Rad) were used as the molecular weight markers. Subsequently, the proteins were transferred to Immuno-Blot^®^ PVDF membrane (1620177, Bio-Rad). After rinsing with MilliQ water twice, the membrane was treated with the antigen pre-treatment solution (SuperSignal Western Blot Enhancer, 46640, ThermoFisher Scientific) according to the manufacturer’s instructions. Anti-cMyc (R950-25, ThermoFisher Scientific) in primary antibody diluent (SuperSignal Western Blot Enhancer, 46640, ThermoFisher Scientific) (1:10 000 dilution) was then used as the primary antibody, while Amersham ECL mouse IgG, horseradish peroxidase (HRP)-linked whole antibodies (from sheep) (NA931-1ML, GE Healthcare Life Sciences) at a 1:20 000 dilution were used as the secondary antibody. Signals were generated using West Femto Maximum Sensitivity Substrate (34095, ThermoFisher Scientific) according to the manufacturer’s instructions and detected using ChemiDoc MP (Bio-Rad).

### Subcellular localization studies

DNA coating and bombardment of onion epidermis were performed by the Biolistic^®^ PDS-1000/He Particle Delivery System (Bio-Rad) according to the manufacturer’s protocol. A total of 0.75 mg of 10 µm gold particles (Bio-Rad) and 1.25 µg of plasmid DNA were used for each bombardment event. Onion epidermal cells were treated with DAPI (D3571, ThermoFisher Scientific) to stain the nuclei and observed using a confocal microscope, Olympus FV1000 [excitation 488 nm, emission 510 nm for green fluorescent protein (GFP); and excitation 405 nm, emission 461 nm for DAPI]. The images were processed by FV10-ASW 4.2 Viewer.

### Yeast transcriptional assays

Yeast transcriptional assays using the Gal4BD system was done according to a previous report (Xiong *et al.*, 2014) in the yeast strain Y2HGold (TaKaRa). The LexA-dependent transcriptional assay was done according to a previous report (Lau *et al.*, 2011) in the yeast strain W303a (constructed by Rodney Rothstein, Columbia University; provided by Professor Ting-Fung Chan, The Chinese University of Hong Kong). The plasmids used, JK1621 and pLGΔ312S (Lau *et al.*, 2011), were from Professor On-Sun Lau, National University of Singapore. pLexA (Addgene plasmid # 11342) was a gift from Guy Caldwell (The University of Alabama). The β-galactosidase assay was done with the overnight culture of yeast grown at 28 °C in SD-Trp-Ura medium (2% glucose) using the Yeast beta-Galactosidase Assay Kit (75768, ThermoFisher Scientific) according to the manufacturer’s instructions. The β-galactosidase activity was detected at 420 nm using a microplate reader (SpectraMax Plus 384 Microplate Reader, Molecular Devices). The absorbance at 420 nm was normalized with the cell density as determined by the absorbance at 600 nm. The β-galactosidase activities of yeast cells carrying both the pLexA and pLGΔ312S plasmids served as the control for basal signals. The β-galactosidase activities of yeast cells carrying both the pLexA and JK1621 plasmids were then normalized to the basal signals.

### Transcriptome analyses and transcription factor-binding motif searches

The total RNA of transgenic soybean hairy roots was extracted using TRIzol™ Reagent (15596026, ThermoFisher Scientific) according to the manufacturer’s protocol. The isolation of total mRNA using oligo(dT) beads, generation of strand-specific libraries, and RNA sequencing were performed by a commercial service (BGI-Hong Kong, Hong Kong). The libraries were sequenced using the Illumina Hi-Seq 4000 paired end platform to generate >80 million raw reads of 101 bases from each library. Cleaned reads were aligned to the reference genome of soybean (version Wm82v2a1) (Schmutz *et al.*, 2010) by HISAT (version 2.0.4) (Kim *et al.*, 2015). The alignment result was then subjected to StringTie (version 1.3.0) (Pertea *et al.*, 2015) for transcript assembly, and the assembled transcripts were used to generate a gene list. Ballgown (Frazee *et al.*, 2015) was used to compute the expression levels of genes and to identify differentially expressed genes among the sequencing libraires. Selected genes were subjected to Gene Ontology (GO) enrichment analyses by agriGO (Tian *et al.*, 2017). All protein-coding genes from the soybean reference genome (version Wm82v2a1) (Schmutz *et al.*, 2010) were regarded as the background for the analyses. A hypergeometric test was performed with the threshold *P*<0.05. After that, hierachical diagrams were generated according to the internal relationships among the GO terms. Genomic sequences 3 kb upstream of the translational start site of putative target genes were extracted from our in-house genome sequence of wild soybean W05. The extracted sequences were subjected to MEME-Suite (version 4.11.3) (Machanick and Bailey, 2011) to discover putative transcription factor-binding motifs (E-value ≤0.05). The best-match motif from the genomic sequence upstream of the translational start site of the putative target gene was then subjected to homology search against known transcription factor-binding motifs by JASPAR (Khan *et al.*, 2018).

### Droplet digital PCR (ddPCR)

The cDNA synthesized by PrimeScript™ RT Master Mix (Perfect Real Time) (RR036Q, TaKaRa) was used for ddPCR with QX200™ ddPCR EvaGreen Supermix (1864034; Bio-Rad) using the QX200™ Droplet Digital™ PCR system (Bio-Rad) according to the manufacturer’s instructions. Primers for ddPCR are listed in Supplementary Table S1.

### EMSA

Glutathione *S*-transferase (GST) alone or GST–GmABAS1 recombinant protein was purified from the *E. coli* strain Rosetta2 transformed with pGEX-4T-1 alone or pGEX-4T-1*-GmABAS1* using the MagneGST™ Protein Purification System (V8600, Promega) according to the manufacturer’s protocol. After protein elution, the protein buffer was changed from the elution buffer to the 2× EMSA buffer (300 mM KCl, 20 mM Tris, pH 7.4) using the Nanosep Device (10K, OD003C34, Pall Corporation). The Qubit™ Protein Assay Kit (Q33211, ThermoFisher Scientific) was used for protein quantitation. DTT, EDTA, and dsDNA probe were then added to the purified protein in the 2× EMSA buffer to make up to a final buffer concentration of 150 mM KCl, 10 mM Tris, 0.1 mM EDTA, and 0.1 mM DTT. All the buffers were supplemented with cOmplete™, EDTA-free Protease Inhibitor Cocktail (5056489001, Merck). The protein–DNA mixture was incubated at room temperature for 20 min before electrophoresis in a 6% acrylamide gel for non-competitive EMSA or a 10% acrylamide gel for competitive EMSA in Tris-borate-EDTA (TBE) buffer. After electrophoresis, the gel of the non-competitive EMSA was stained with SYBR^®^ Green (S7563, ThermoFisher Scientific) and then documented by the Gel Doc™ EZ Gel Documentation System (Bio-Rad), while the gel of the competitive EMSA was directly subjected to documentation by the Gel Doc™ EZ Gel Documentation System (Bio-Rad). Sequences of the DNA probes are listed in Supplementary Table S1. The 6-FAM-labelled double-stranded probes were ordered directly from Integrated DNA Technologies, while the individual single-stranded oligos for unlabelled double-stranded probes were ordered from ThermoFisher Scientific. To prepare double-stranded unlabelled DNA probes, each pair of DNA oligos was incubated at 100 °C for 5 min, followed by incubation for 30 s at a temperature 5 °C lower than the previous step using a thermal cycler (ramp rate: 0.1 °C s^–1^).

### Yeast promoter-binding reporter assay

The yeast promoter-binding reporter assay was done according to a previous report (Alves *et al.*, 2011). The sequence 3 kb upstream of the translational start of *Glyma.01G060300* was cloned upstream of the *LacZ* gene in pLacZi (TaKaRa, a gift from Professor King-Lau Chow of The Hong Kong University of Science and Technology) at the *Sma*I site. Empty pLacZi or pLacZi containing the promoter of *Glyma.01G060300* were digested at the *Nco*I site before each being integrated into the genome of yeast strain W303α. The transformation was done using the LiAc method according to the Yeast Protocols Handbook (TaKaRa). After that, the yeast strain W303α was further transformed with pBEVY-T empty vector (Miller *et al.*, 1998), pBEVYT-*cMyc-GmABAS1*, or pBEVY-T-*cMyc-GmABAS1∆* using the LiAc method according to the Yeast Protocols Handbook (TaKaRa). The β-galactosidase assay was done using the overnight culture of yeast grown at 28 °C in SD-Trp-Ura medium (with 2% fructose) utilzing the Yeast beta-Galactosidase Assay Kit (75768, ThermoFisher Scientific) according to the manufacturer’s instructions. The β-galactosidase activity was detected at 420 nm using a microplate reader (SpectraMax Plus 384 Microplate Reader, Molecular Devices). The absorbance at 420 nm was normalized with the cell density as determined by the absorbance at 600 nm. The β-galactosidase activities of yeast cells carrying both the pLacZi and the plasmid, empty pBEVY-T (Miller *et al.*, 1998), pBEVY-T-*cMyc-GmABAS1*, or pBEVY-T-cMyc-*GmABAS1Δ*, served as the control for basal signals. The β-galactosidase activities of yeast cells carrying both pLacZi containing the promoter of *Glyma.01G060300* and the plasmid, empty pBEVY-T (Miller *et al.*, 1998), pBEVY-T-*cMyc-GmABAS1*, or pBEVY-T-*cMyc-GmABAS1Δ*, were then normalized to their respective basal signals.

### Plant materials and treatments

For the osmotic stress treatment of soybean plants, seeds were first germinated in vermiculite for 1 week before being transferred to 1/2× Hoagland’s solution (Hoagland and Arnon, 1950). After the emergence of the first trifoliates, the plants were treated with 300 mM mannitol in 1/2× Hoagland’s solution for 0, 4, or 24 h. The experiment was performed twice. In each replicate, at least three plants were pooled as one sample.

To obtain transgenic hairy roots from soybean, seeds were washed with 5% (v/v) household bleach for 5 min with shaking and then rinsed at least five times with MilliQ water. The seeds with crumpled teguments were placed on wet cotton soaked with MilliQ water in a plastic box in the dark at 28 °C for 48 h for germination. Germinated seeds with emerged radicles were selected for transformation. Selected seeds were placed in MilliQ water and the two cotyledons were separated. After the removal of teguments by a surgical blade, cotyledons were left in 50 mM l-cysteine. Then *Agrobacterium rhizogenes* K599 transformed with pCambia3301 empty vector or pCambia3301 with the phosphinothricin resistance gene [downstream of a *Cauliflower mosaic virus* (CaMV) 35S promoter] replaced by *cMyc-GmABAS1*, prepared as described (Estrada-Navarrete *et al.*, 2007), was injected into the cotyledons at the convex side using a 1 ml syringe. Excess bacteria on the surface of the infected cotyledons were removed by a quick rinse with MilliQ water followed by patting dry on filter paper. The infected cotyledons were then transferred to Petri dishes containing sterile 0.7% (w/v) water agar. After that, the Petri dishes were sealed with parafilm and incubated under a photoperiod of 16 h/8 h (light/dark) for 3 weeks. Callus formation was observed 1 week after the infection. Each root represented one individual transformation event. After the appearance of the callus, the hairy roots were allowed to grow for a further 2 weeks. Cotyledons bearing the hairy roots were placed in either 1/2× Hoagland’s solution (Hoagland and Arnon, 1950) supplemented with 100 µM ABA in 0.1% (v/v) methanol, or in the same medium without ABA (mock treatment), for 5 h under light. During the treatment, the hairy roots were placed face-down in the solution. After the treatment, each hairy root was harvested separately, with a small segment of each root cut and subjected to β-glucuronidase (GUS) staining to identify successful transformants. The remaining hairy root samples were frozen in liquid nitrogen and stored at –80 °C for RNA extraction. GUS staining was done according to a previous report (Kereszt *et al.*, 2007). The successfully transformed roots were then used for RNA extraction. The experiment was performed twice. In each replicate; transgenic roots from at least three cotyledons were pooled as one sample for RNA extraction.

Transgenic *A. thaliana* (Col-0) expressing *GmABAS1* or *GmABAS1Δ* were generated using the *Agrobacterium*-mediated transformation method (Bechtold *et al.*, 1993). Seed germination assay and stomatal aperture assay of *A. thaliana* were done according to a previous report (Burnette *et al*., 2003). For seed germination assay, seeds of *A. thaliana* were surface-sterilized using absolute household bleach for 3 min followed by rinsing with sterile water three times. After that, the seeds were transferred onto 1/2× Murashige and Skoog (MS) medium with 1% sucrose and 0.9% agar with or without 4 µM ABA in 0.1% (v/v) methanol. The seeded agar plates were incubated at 4 °C in the dark for 2 d before placing them under continuous light at 25 °C for 11 d. For stomatal aperture assay of *A. thaliana*, rosette leaves of 4-week-old plants grown on soil were detached and placed on a perfusion solution (50 mM KCl, 10 mM MES, pH 7.0) (Burnette *et al*., 2003; Ogata *et al.*, 2017) under light for 2 h. After that, the leaves were transferred to the perfusion solution with 100 µM ABA in 0.1% (v/v) methanol or 0.1% (v/v) methanol only (mock) under light for another 2 h. After the treatment, the lower epidermis was peeled off and observed under a light microscope (Eclipse 80i, Nikon) to obtain differential interference contrast (DIC) images. The stomatal aperture was measured by a digital ruler (SPOT Advanced, version 4.6, Diagnostic Instruments, Inc.). The same treatment was done to the rosette leaves of 4-week-old *A. thaliana* for gene expression studies.

### Statistical analyses

Statistical Package for Social Sciences (version 16.0; SPSS Inc., Chicago, IL, USA) was used for statistical analyses.

## Results

### *GmABAS1* encodes a 1R-subtype MYB

In a survey of genes close to the salt tolerance QTL on soybean chromosome 3 (Qi *et al.*, 2014), we have identified a gene encoding a 1R-subtype MYB in the wild soybean parent of a recombinant inbred population, with a corresponding 19 bp deletion mutant in the cultivated parent. We named this gene *GmABAS1* (*Glycine max ABA-Sensitive 1*) (GenBank accession no. MH788966) due to the putative associations with ABA sensitivity of the 19 bp deletion mutant, *GmABAS1∆* (GenBank accession no. MH788967). Based on our resequencing data of 31 soybean accessions (Qi *et al.*, 2014), which were later confirmed by Sanger sequencing, we found that all the wild, and most of the cultivated, soybean accessions tested contain the intact *GmABAS1* gene, while a few other cultivated soybean accessions possess a truncated version with the 19 bp deletion (at +10 to +28 nt from the translational start site), which we named *GmABAS1∆* (Supplementary Fig. S1).

**Fig. 1.**
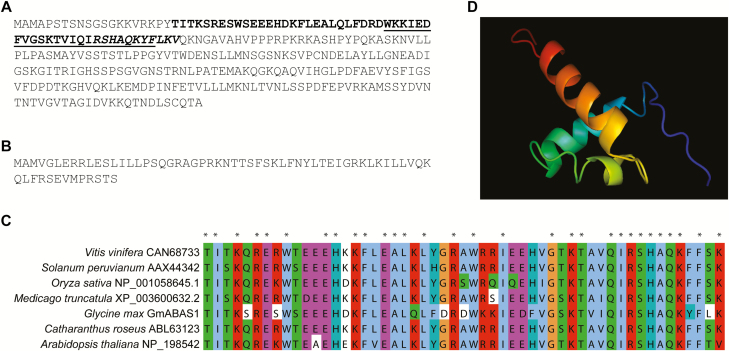
*GmABAS1* encodes a 1R-subtype MYB. Translated amino acid sequence of (A) the intact GmABAS1 and (B) the truncated GmABAS1∆. Bold, helix–turn–helix (HTH) MYB-type domain; underlined, HTH motif; italic, nuclear localization signal. (C) Alignment of the HTH domain of 1R-subtype MYBs from *Vitis vinifera* CAN68733, *Medicago truncatula* XP_003600632.2, *Solanum peruvianum* AAX44342, *Catharanthus roseus* ABL63123, *Oryza sativa* NP_001058645, and *Arabidopsis thaliana* NP_198542 (Jia *et al.*, 2009) with *Glycine max* GmABAS1 (this work). *indicates conserved amino acids. (D) The ribbon structure of GmABAS1 as predicted by the Phyre2 software. Red to purple, N-terminus to C-terminus.

**Fig. 2.**
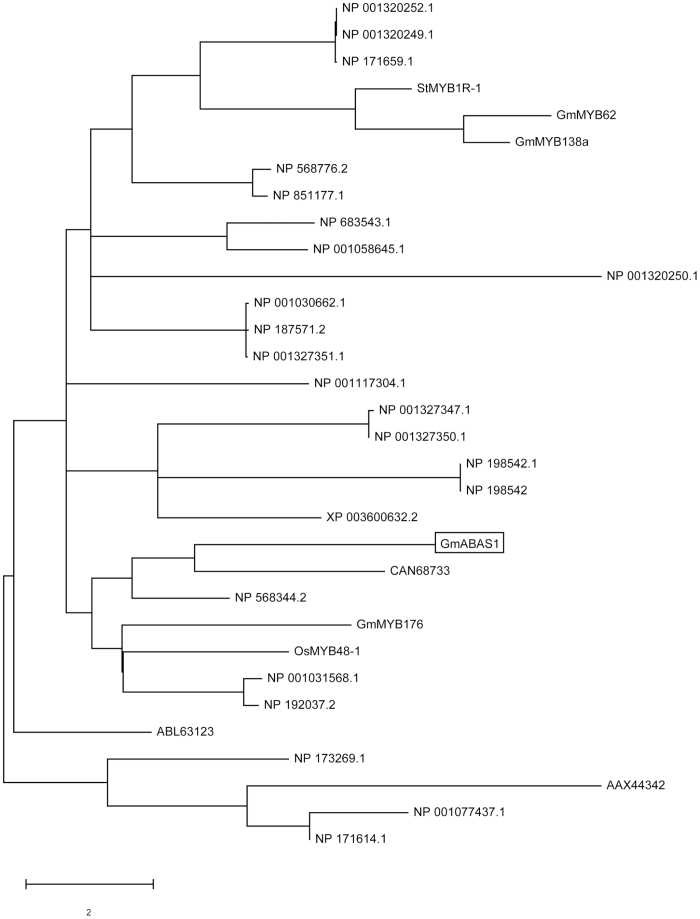
Phylogenetic study of GmABAS1, selected homeodomain-like proteins from *A. thaliana* and 1R MYB proteins from plants. The amino acid sequences of the proteins were aligned for the construction of the phylogenetic tree using MEGA7. Homeodomain-like proteins from *A. thaliana*: NP_187571.2, NP_001327351.1, NP_001030662.1, NP_001327347.1, NP_001327350.1, NP_568776.2, NP_851177.1, NP_171659.1, NP_001031568.1, NP_192037.2, NP_001320252.1, NP_001320249.1, NP_001320250.1, NP_198542.1, NP_001077437.1, NP_171614.1, NP_568344.2, NP_173269.1, NP_683543.1, and NP_001117304.1. Proteins from various plant species which were found to contain the 1R MYB domain: CAN68733 (from *Vitis vinifera*), XP_003600632.2 (from *Medicago truncatula*), AAX44342 (from *Solanum peruvianum*), ABL63123 (from *Catharanthus roseus*), NP_001058645.1 (from *Oryza sativa*), and NP_198542 (from *A. thaliana*). Characterized 1R MYB proteins from various plant species: StMYB1R-1, OsMYB48-1, GmMYB176, GmMYB62, and GmMYB138a. GmABAS1 is enclosed in a box.

Sequence analyses showed that *GmABAS1* encodes a MYB-type HTH domain (Fig. 1A, D) and the nuclear localization signal (Fig. 1A). The amino acid sequence of the HTH domain shares consensus with other plant 1R-subtype MYBs (Fig. 1C). On the other hand, *GmABAS1∆* contains a premature translational stop and encodes a truncated protein that loses both the MYB-type HTH domain and the nuclear localization signal (Fig. 1B). The predicted ribbon protein structure of GmABAS1 by Phyre2 showed the HTH domain (Fig. 1D). An alignment of the translated sequence of GmABAS1 with peptide sequences of the model organism *A. thaliana* by BLASTX (Gish and States, 1993) showed that GmABAS1 shares homology with homeodomain-like proteins. However, when GmABAS1 was then subjected to phylogenetic analysis with homeodomain-like proteins and other 1R MYB proteins in plants, the results showed that GmABAS1 does not cluster with any of the characterized 1R MYB proteins (Fig. 2). This indicates that GmABAS1 is a novel member of 1R MYB proteins.

**Fig. 3.**
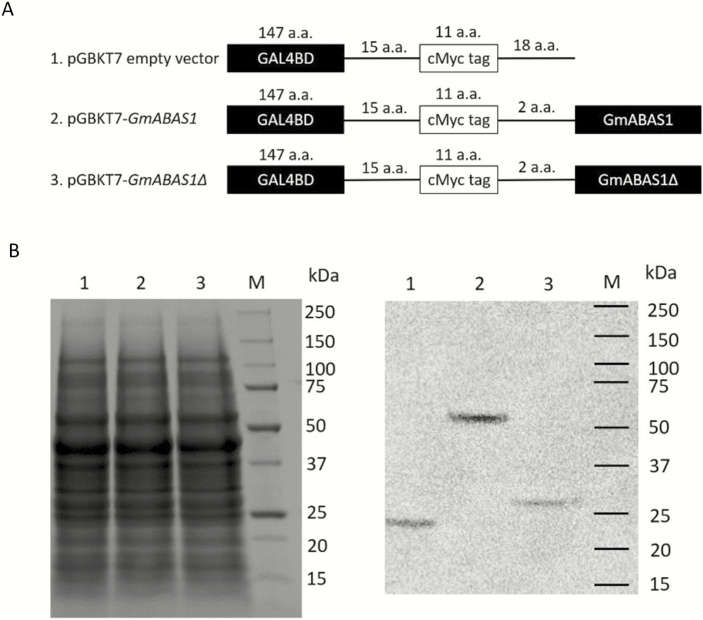
Size determination of the translation products of *GmABAS1* and *GmABAS1∆*. (A) Schematics of the plasmid constructs for transformation into the yeast strain Y2HGold. (B) Total proteins from the transgenic yeast strains on SDS–PAGE. (C) Western blot detected by anti-cMyc antibodies. 1, pGBKT7 empty vector; 2, pGBKT7-*GmABAS1*; 3, pGBKT7-*GmABAS1∆*; M, molecular weight markers.

To demonstrate the premature translational stop of *GmABAS1∆*, GmASAS1 and GmABAS1∆ were expressed in the yeast strain Y2HGold for the detection of the protein sizes. Results from the western blot confirmed the predicted sizes of the GAL4BD-cMyc-GmABAS1 and the GAL4BD-cMyc-GmABAS1∆ fusion proteins at ~51 kDa and ~26 kDa, respectively (Fig. 3). In the subsequent experiments, GmABAS1Δ was used as a negative control.

### GmABAS1 exhibited transcriptional repressor activities

**Fig. 4.**
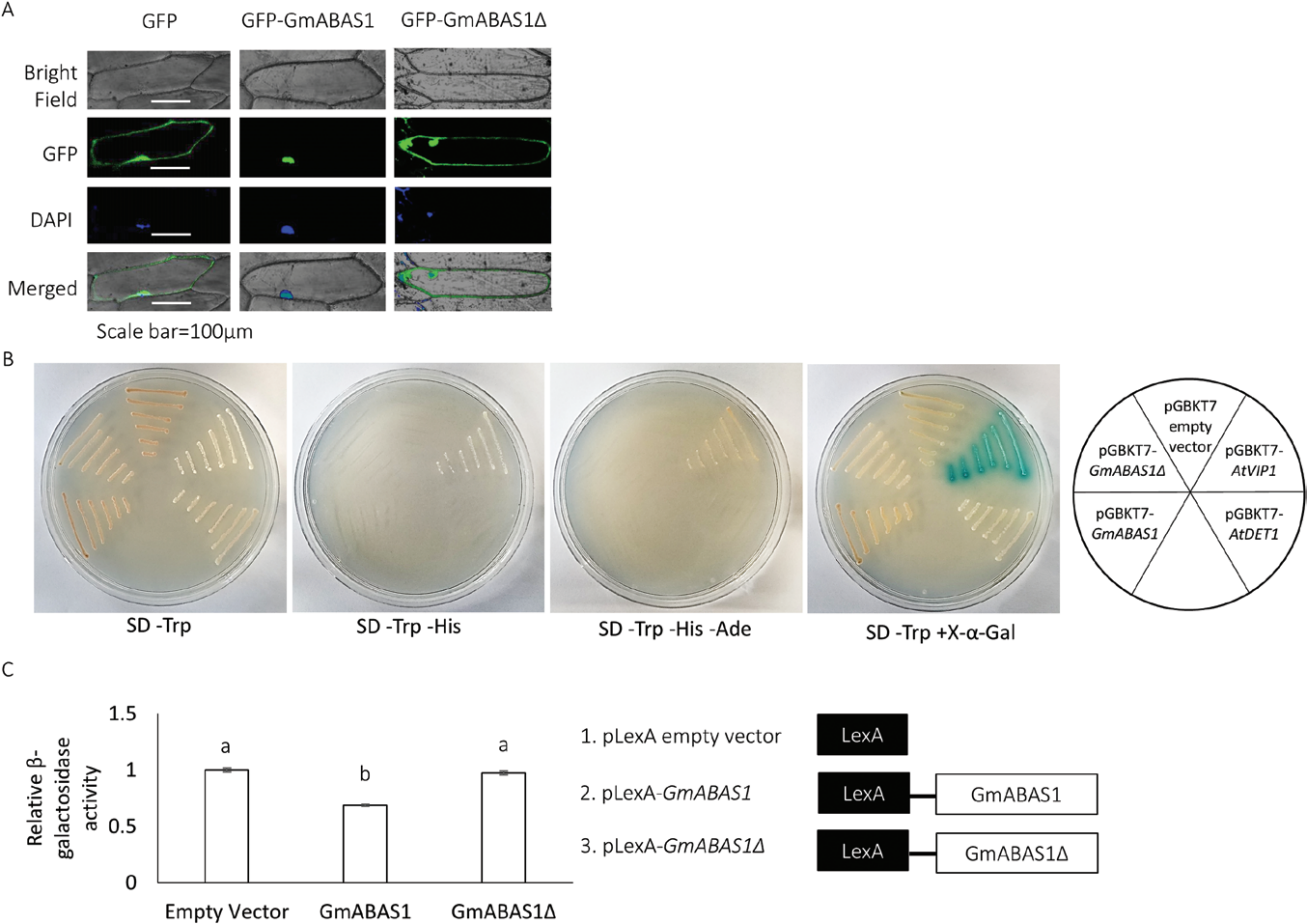
GmABAS1 exhibited transcriptional repressor activities. (A) Detection of the subcellular localization of GmABAS1 and GmABAS1∆ using GFP fusion proteins. GFP was fused to the N-terminus of GmABAS1 or GmABAS1∆, and the fusion construct was cloned into the plasmid V7, downstream of a CaMV 35S promoter. The plasmid DNA was coated onto gold particles which were then bombarded into onion epidermal cells. DAPI was used to stain the nucleus. Green fluorescence signals were analysed as described in the Materials and methods. For each construct, all cells giving the GFP signal showed the same pattern. The experiment was performed twice with similar results. (B) Transactivation assay using the yeast strain Y2HGold. *GmABAS1*, *GmABAS1∆*, *AtVIP1* (a transcriptional activator, positive control), or *AtDET1* (a transcriptional repressor, negative control) was cloned into the plasmid pGBKT7 to produce Gal4BD fusion proteins. The transgenic yeast cells were selected by SD-Trp medium. Metabolic markers *HIS3*, *ADE2*, and *MEL1* were used to report transactivation activities. The transgenic yeast cells expressing *Gal4BD-GmABAS1* or *Gal4BD-GmABAS1∆* could not grow on medium without His and/or Ade. The transgenic yeast cells also could not hydrolyse X-α-Gal. The experiment was performed twice with similar results. (C) Transcriptional repression assay using the yeast strain W303a. *GmABAS1* or *GmABAS1∆* was cloned into the plasmid pLexA which was then transformed into W303a. The transgenic yeast was then co-transformed with either the JK1621 plasmid carrying the *LacZ* gene, driven by the minimal promoter *CYC1* downstream of the LexA-binding sites, or with the pLGΔ312S plasmid without the LexA-binding sites. The β-galactosidase activities of the yeast co-transformed with JK1621 and pLexA plasmids were normalized by the activities of the yeast co-transformed with pLGΔ312S and the corresponding pLexA plasmids. Error bars represent SEs of three technical repeats. Each lower case letter above the bars indicates a statistically distinct group with *P*<0.05 based on one-way ANOVA followed by Tukey HSD test. The experiment was performed twice with similar results.

Localization in the nucleus is a common characteristic of transcription factors. To visualize the subcellular localization of GmABAS1, a GFP fusion protein construct was transiently expressed in onion epidermis by particle bombardment. The results showed that GFP–GmABAS1 localized in the nucleus while the truncated GFP–GmABAS1∆ exhibited diffused signals similar to GFP alone (Fig. 4A).

Yeast transcriptional assays were then carried out to study the regulatory function of the GmABAS1 protein. *GmASAS1* and *GmABAS1∆* were cloned into the plasmid pGBKT7 to produce Gal4BD fusion proteins in the yeast strain Y2HGold (Fig. 4A). *HIS3*, *ADE2*, and *MEL1*, downstream of the GAL4-binding site, were used as the reporters. *AtVIP1* (Tsugama *et al.*, 2012), a characterized transcriptional activator, was cloned into the plasmid pGBKT7 as the positive control, while *AtDET1* (Lau *et al.*, 2011), a characterized transcriptional repressor, was employed as the negative control. Results showed that neither Gal4BD–GmABAS1 nor Gal4BD–GmABAS1∆ could activate transcription (Fig. 4B).

We therefore looked in the opposite direction and tested whether GmABAS1 was a transcriptional repressor, using the LexA operator-dependent reporter system (Lau *et al.*, 2011). In this experiment, the plasmid JK1621 containing the *lacZ* gene downstream of the LexA-binding site was used as a reporter. In the negative control experiment, the plasmid pLGΔ312S containing the *lacZ* reporter gene without the upstream LexA-binding site was used. Results showed that the expression of LexA–GmABAS1 led to a significant reduction of the β-galactosidase activity, compared with LexA–GmABAS1∆ and LexA only (Fig. 4C). This demonstrated the function of GmABAS1 as a transcriptional repressor, while the transcriptional regulatory function was lost in GmABAS1Δ.

**Fig. 5.**
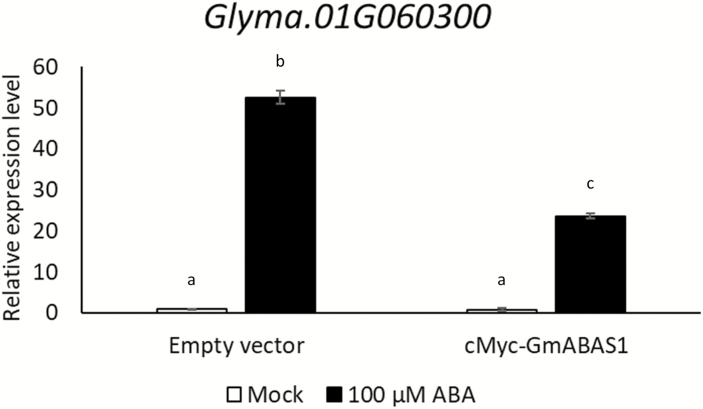
The expression levels of *Glyma.01G060300* in the presence of *GmABAS1* and ABA treatment. The copy numbers of mRNAs of *Glyma.01G060300* were determined using digital droplet PCR (ddPCR) and normalized to that of the *60S ribosomal protein* gene (Le *et al.*, 2012). The error bar represents the SE of three technical repeats. Each lower case letter above the bar indicates a statistically distinct group with *P*<0.05 based on one-way ANOVA followed by a least significant difference (LSD) test. Mock: no ABA treatment. The experiment was performed twice with similar results.

As mentioned in the Introduction, some 1R-subtype MYBs are closely related to ABA signalling. To identify the putative gene targets of GmABAS1 under ABA treatment, *cMyc-GmABAS1* driven by the CaMV 35S promoter was transformed into soybean hairy roots induced from the cotyledons of soybean accession W05. The soybean hairy roots were subjected to 100 µM ABA or mock treatment for 5 h. After ABA treatment, the hairy roots were harvested one by one. A small segment of each root was subjected to GUS staining for the screening of successful transformants, while the remaining segment was frozen in liquid nitrogen and stored in –80 °C for RNA extraction and RNA sequencing. Using the empty vector transformants as the negative control, a set of ABA-induced genes were found to have lower fold induction when *cMyc-GmABAS1* was overexpressed (Supplementary Table S3). These genes were regarded as the potential targets of GmABAS1. In terms of molecular functions, the potential target genes were enriched in GO terms corresponding to DNA binding and transcription factor activity (Supplementary Fig. S2A). Regarding biological processes, GO terms corresponding to the regulation of transcription were also enriched (Supplementary Fig. S2B). Among the potential targets of GmABAS1 (Supplementary Table S3), six genes were found to be ABA sensitivity related based on the functional annotation (Table 1). In the RNA-seq data set, these candidate genes were shown to be induced by ABA treatment. However, when *cMyc-GmABAS1* was overexpressed, the fold induction was decreased (Table 1). The expression patterns of these candidate genes were validated by ddPCR. *Glyma.01G060300*, which is annotated as *ABI five-binding protein 3* (*AFP3*), showed a significant induction by ABA, but the fold induction was lowered significantly when *GmABAS1* was overexpressed under similar ABA treatment (Fig. 5).

**Table 1.** Fold induction of selected ABA sensitivity-related genes based on transcriptome analyses

ABA sensitivity-related gene	Functional annotation	Fold induction by ABA treatment in empty vector control (A)^*a*^	Fold induction by ABA treatment in *cMyc-GmABAS1* expressers (B)^+^	Relative fold repression due to *cMyc-GmABAS1* (A/B)
*Glyma.19G069200*	Highly ABA-induced PP2C gene 3	148.56	55.80	2.66
*Glyma.01G060300*	ABI five-binding protein 3	57.28	22.68	2.53
*Glyma.18G267200*	ABI five-binding protein 3	9.46	5.81	1.63
*Glyma.05G227100*	Protein phosphatase 2C family protein	38.86	27.46	1.42
*Glyma.06G204100*	ABI five-binding protein 3	79.66	128.01	0.62
*Glyma.14G066400*	Protein phosphatase 2CA	13.01	10.85	1.20

^*a*^ Average of two biological repeats

**Fig. 6.**
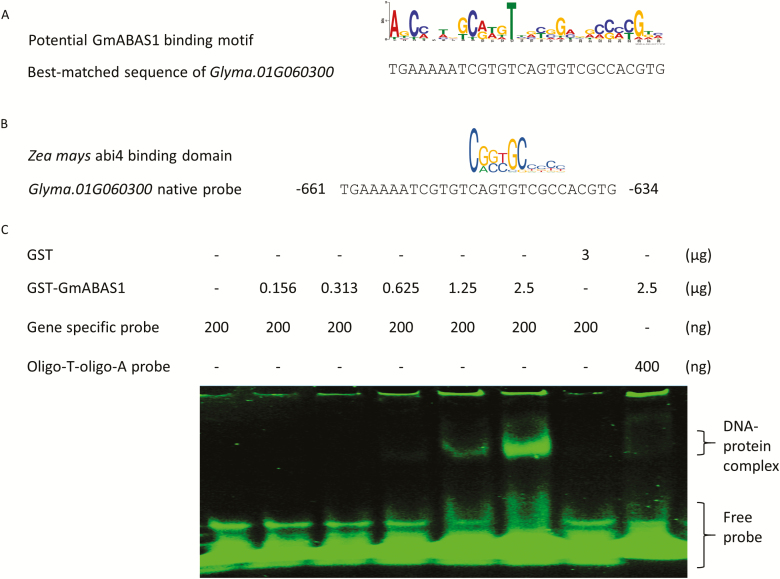
EMSA showing the binding of GmABAS1 to the promoter region of *Glyma.01G060300*. (A) Potential DNA motifs targeted by GmABAS1 as predicted from sequences 3 kb upstream of the translational start sites of the potential target genes of GmABAS1 (Supplementary Table S3) and the best-matched sequence upstream of the translational start site of *Glyma.01G060300*. (B) The best-matched sequence of *Glyma.01G060300* is from 661 to 634 bases upstream of the translational start site. The motif sharing homology with the *Zea mays* ABI4-binding domain was mutated. (C) EMSA demonstrating the binding between GmABAS1 and the promoter region of *Glyma.01G060300*. GmABAS1 was purified with a GST tag. Purified GST alone or GST–GmABAS1 was incubated with gene-specific or oligo(T)–oligo(A) dsDNA probes. The incubated protein–DNA probe mixture was electrophoresed in a 6% acrylamide gel which was then stained by SYBR^®^ Green EMSA nucleic acid gel stain. Retardation of the DNA probe migration was due to binding with GmABAS1.

To search for the potential DNA-binding motif of GmABAS1, the sequences 3 kb upstream of the translational start sites of the potential targets (Supplementary Table S3), predicted from the genome sequence of soybean accession W05 (Xie *et al.*, 2019), were subjected to transcription factor-binding motif analysis by MEME-ChIP (Machanick and Bailey, 2011). Using these sequences, a potential transcription factor-binding motif was found (Fig. 6A). With this motif as a template, a sequence that best matched the predicted GmABAS1-binding motif was found upstream of the translational start site of *Glyma.01G060300*. Based on the genome sequence of soybean accession W05 (Xie *et al.*, 2019), this motif is located at the possible promoter region of *Glyma.01G060300* (Fig. 6B). The motif from *Glyma.01G060300* was further subjected to homology search against known transcription factor-binding motifs by JASPAR (Khan *et al.*, 2018). The search result showed that the motif from the promoter region of *Glyma.01G060300* shared homology with the ABI4-binding motif from *Zea mays* (Fig. 6B). Based on these results, it is possible that the motif from the promoter region of *Glyma.01G060300* is a binding site of GmABAS1.

The sequence containing the predicted GmABAS1-binding motif in the promoter region of *Glyma.01G060300* (Fig. 6B) was used for generating dsDNA probes for EMSA to confirm whether this is the binding site of GmABAS1. GST–GmABAS1 fusion protein was purified and incubated with the dsDNA probes. The binding of GmABAS1 to the motif from *Glyma.01G060300* was demonstrated by a retardation of the probe signal migration compared with the free probes (Fig. 6C). GST alone and dsDNA probes composed of oligo(T) and oligo(A) were employed as the negative controls to show the specificity of the protein–DNA interaction. When the probe comprising the predicted GmABAS1-binding motif was incubated with GST alone, there was no shifted signal. When GST–GmABAS1 was incubated with the oligo(T)–oligo(A) probe, the shifted signal was much weaker than that with the probe containing the predicted GmABAS1-binding motif (Fig. 6C).

To further confirm the binding of GmABAS1 to the motif from *Glyma.01G060300*, competitive EMSA was performed (Supplementary Fig. S3). In the competitive EMSA, unlabelled native probe or mutated probe was employed as the competitor (Supplementary Fig. S3A). For the mutated probe, the sequence sharing homology with the ABI4-binding motif from *Z. mays* was mutated (Supplementary Fig. S3A). The binding of GmABAS1 to the motif from *Glyma.01G060300* was observed (Supplementary Fig. S3B). GST alone was employed as the negative control to show the specificity of the protein–DNA interaction. When the 6-FAM-labelled probe was incubated with GST alone, there was no shifted signal (Supplementary Fig. S3B). When the unlabelled mutated probe was used as the competitor, the shifted signal was stronger than when the same amount of unlabelled native probe was used as the competitor (Supplementary Fig. S3B). This result indicates the weakened ability of the mutated probe to bind to GmABAS1 and demonstrates the sequence specificity of the binding between the native probe and GmABAS1.

**Fig. 7.**
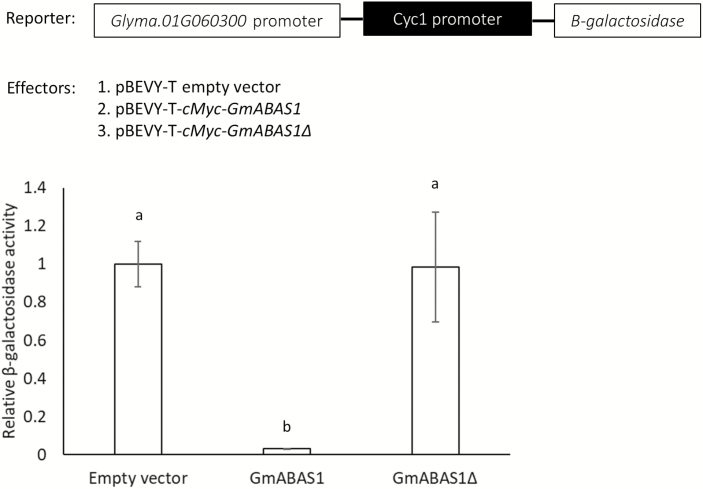
Yeast promoter-binding reporter assay showing the binding of GmABAS1 to the promoter of *Glyma.01G060300* and the transcriptional repressor activity of GmABAS1. The sequence 3 kb upstream of the translational start of *Glyma.01G060300* was cloned upstream of the *LacZ* gene in pLacZi. This construct was used as the reporter. The effectors were pBEVY-T empty vector, pBEVY-T-*cMyc-GmABAS1*, or pBEVY-T-*cMyc-GmABAS1Δ*, respectively. The yeast strain W303α was co-transformed with the reporter and the effector plasmids. The β-galactosidase assay was done using the overnight culture of yeast grown at 28 °C in SD-Trp-Ura medium (with 2% fructose). The β-galactosidase activity was detected at 420 nm using a microplate reader (SpectraMax Plus 384 Microplate Reader, Molecular Devices). The absorbance at 420 nm was normalized with the cell density as determined by the absorbance at 600 nm. The β-galactosidase activities of yeast cells carrying both pLacZi without the promoter of *Glyma.01G060300* and either empty pBEVY-T, pBEVY-T-*cMyc-GmABAS1*, or pBEVY-T-cMyc-*GmABAS1Δ* served as the control for basal signals. The β-galactosidase activities of yeast cells carrying both pLacZi containing the promoter of *Glyma.01G060300* and either empty pBEVY-T, pBEVY-T-*cMyc-GmABAS1*, or pBEVY-T-*cMyc-GmABAS1Δ* were then normalized to their respective basal signals. Each error bar represents the SE of three technical repeats. Each lower case letter above the bar indicates a statistically distinct group with *P*<0.05 based on one-way ANOVA followed by Tukey HSD test. The experiment was performed twice with similar results.

The binding of GmABAS1 to the promoter of *Glyma.01G060300* and the transcriptional repressor activity of GmABAS1 was further confirmed using the yeast promoter-binding reporter assay (Fig. 7). In the assay, LacZ (β-galactosidase) downstream of the promoter of *Glyma.01G060300* was used as the reporter. When cMyc-GmABAS1 was used as the effector, the activity of β-galactosidase was reduced compared with the empty vector control and the cMyc-GmABAS1Δ control (Fig. 7). This result indicates the binding of GmABAS1 to the promoter of *Glyma.01G060300* and the transcriptional repressor activity of GmABAS1.

**Fig. 8.**
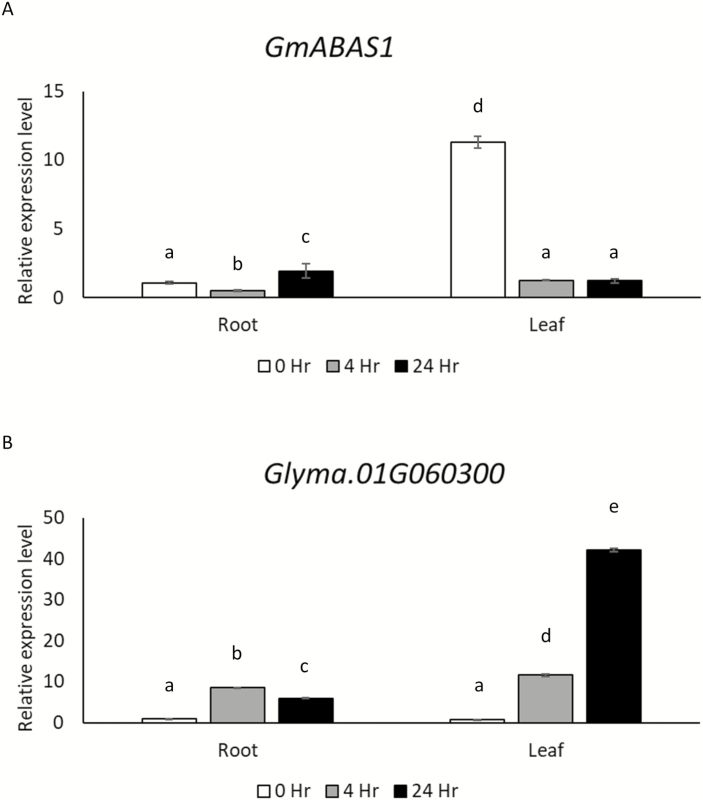
Osmotic stress-induced expression profiles of *GmABAS1* and *Glyma.01G060300*. Soybean seedlings of W05 were treated with 300 mM mannitol in 1/2× Hoagland’s solution to induce osmotic stress for 0, 4, or 24 h respectively. Total cDNA was synthesized from total RNA for ddPCR. The copy numbers of mRNA of (A) *GmABAS1* and (B) *Glyma.01G060300* were normalized to that of *ELF-1b* (Yim *et al.*, 2015). The error bar represents the SE of three technical repeats. Each lower caase letter above the bar indicates a statistically distinct group with *P*<0.05 based on one-way ANOVA followed by a least significant difference (LSD) test. The experiment was performed twice with similar results.

To further demonstrate the relationships between the expression patterns of *GmABAS1* and *Glyma.01G060300 in planta*, soybean (W05) seedlings were treated with 300 mM mannitol to induce osmotic stress for 0, 4, and 24 h, respectively. When treated with osmotic stress, the expression level of *GmABAS1* in the root was down-regulated at 4 h of mannitol treatment and then up-regulated at 24 h (Fig. 8A). A reciprocal pattern in the expression of *Glyma.01G060300* was observed, in which the expression of *Glyma.01G060300* was up-regulated at 4 h and then down-regulated at 24 h (Fig. 8A). In the leaf, when treated with osmotic stress, the expression of *GmABAS1* was repressed at both 4 h and 24 h (Fig. 8B). In contrast, the expression of *Glyma.01G060300* was elevated in the leaf under osmotic stress at both 4 h and 24 h (Fig. 8B). Altogether, the expression pattern of *Glyma.01G060300* in hairy roots under ABA treatment when *GmABAS1* was overexpressed, the EMSA results, the yeast transcriptional assays and promoter-binding reporter assay, and the reciprocal expression patterns between *GmABAS1* and *Glyma.01G060300* under osmotic stress support the role of GmABAS1 as a transcriptional repressor of *Glyma.01G060300*.

### GmABAS1 enhanced ABA sensitivity

**Fig. 9.**
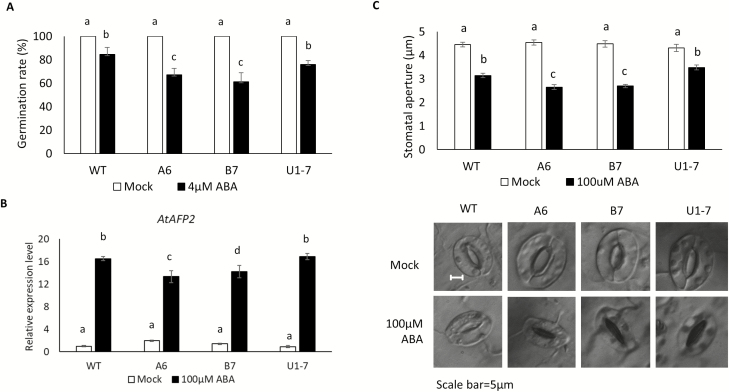
GmABAS1 enhanced ABA sensitivity in transgenic *A. thaliana*. Transgenic *A. thaliana* ectopically expressing *GmABAS1* (A6, B7) or *GmABAS1∆* (U1-7) was subjected to: (A) germination assay and (C) stomatal aperture assay. (A) The seed germination rate at day 5 after 4 µM ABA treatment or mock treatment. The germination rate was calculated from the average of three biological repeats, with >100 seeds from each line recorded. WT, wild type; A6 and B7, *GmABAS1* overexpressers; U1-7, *GmABAS1∆* overexpresser. The error bar represents the SD of three biological repeats. Each lower case letter above the bar indicates a statistically distinct group with *P*<0.05 based on one-way ANOVA followed by a least significant difference (LSD) test. (B) The expression levels of *AtAFP2* in the presence of *GmABAS1* or *GmABAS1Δ* under ABA treatment. Detached leaves of *A. thaliana* were treated with 100 µM ABA or without ABA (mock) in the perfusion solution. The copy numbers of mRNAs of *AtAFP2* (Lynch *et al*., 2017) were determined using digital droplet PCR (ddPCR) and normalized to that of *AtTUBβ* (At5g62690). The error bar represents the SE of three technical repeats. Each lower case letter above the bar indicates a statistically distinct group with *P*<0.05 based on one-way ANOVA followed by a least significant difference (LSD) test. Mock, no ABA treatment. (C) Stomatal aperture assay of transgenic *A. thaliana*. Detached leaves of *A. thaliana* were treated with 100 µM ABA or without ABA (mock) in the perfusion solution. At least 30 stomata from each line were measured. Each error bar represents the SE of all cells measured. ANOVA with Games–Howell post-hoc test was used for statistical analyses. Each lower case letter above the bar indicates a statistically distinct group with *P*<0.05. The experiment was performed twice with similar results.

To test the effect of GmABAS1 on ABA sensitivity, transgenic *A. thaliana* ectopically expressing either *GmABAS1* or *GmABAS1∆* was subjected to seed germination test under ABA treatment. The expression of *GmABAS1* enhanced the sensitivity to ABA during germination, as reflected by the lower germination rates of the *GmABAS1* transgenics than the wild type or the *GmABAS1Δ* transgenics (Fig. 9A). In Arabidopsis, it has been reported that the mutation of *AFP1* and *AFP2* enhanced ABA sensitivity (Lopez-Molina *et al.*, 2003; Lynch *et al*., 2017). The expression of *AtAFP1* and *AtAFP2* was first screened by quantitative real-time PCR (qRT-PCR), and only the expression of *AtAFP1* was not significantly affected by *GmABAS1* under ABA treatment (Supplementary Fig. S4). The effect of *GmABAS1* on the expression of *AtAFP2* was further confirmed using ddPCR. Under ABA treatment, the expression of *AtAFP2* was down-regulated by the overexpression of *GmABAS1* but not by that of *GmABAS1Δ* (Fig. 9B). The down-regulation of *AtAFP2* expression and the enhanced sensitivity to ABA in the germination assay are consistent with the findings from previous reports (Lopez-Molina *et al.*, 2003; Lynch *et al*., 2017). To further investigate the physiological significance of GmABAS1 under ABA treatment, a stomatal aperture assay was conducted, where the overexpression of *GmABAS1*, but not that of *GmABAS1Δ*, led to significantly smaller stomatal apertures under ABA treatment (Fig. 9C). This result is also consistent with the enhanced ABA sensitivity due to the overexpression of *GmABAS1* in the germination assay.

## Discussion

Up till now, the transcriptional regulation functions of 1R-subtype MYB transcription factors in soybean have only received limited attention. This study identified and characterized *GmABAS1* which is the first reported 1R-subtype MYB transcriptional repressor gene in soybean. Despite sharing the 1R-subtype MYB domain with other characterized 1R MYB proteins in plants, GmABAS1 did not cluster with any of these proteins in phylogenetic analysis (Fig. 2). This reveals that GmABAS1 is a novel member of the 1R-subtype MYB proteins in plants. We also demonstrated that GmABAS1 functions as a transcriptional repressor in this study. The intact *GmABAS1* gene exists in all 17 wild and 10 of the 14 cultivated soybeans we have investigated (Supplementary Fig. S1). However, a few cultivated accessions contain a 19 bp deletion mutation (*GmABAS1∆*), which lacks the HTH domain and the nuclear localization signal. *GmABAS1Δ* was therefore used as a loss-of-function negative control in this study (Fig. 3). Although a few 1R-subtype MYB-like proteins have been reported to be associated with ABA sensitivity, the transcriptional regulatory functions and the regulatory targets of these proteins are not clear. In this report, we demonstrate the transcriptional repressor function of GmABAS1 and identify *Glyma.01G060300*, an annotated *AFP3*, as its transcriptional regulatory target. Using Arabidopsis as the model, we also demonstrate the involvement of GmABAS1 in regulating the expression of Arabidopsis *ABI5 binding protein 2* (*AtAFP2*) and ABA sensitivity.

To identify genes that are regulated by GmABAS1 in soybean, the transgenic hairy root system was employed for transcriptome analyses. To facilitate this study, we developed a modified protocol for the generation of transgenic hairy roots using cotyledons. The roots grown from the callus after infection by *A. rhizogenes* could be easily separated from each other, allowing the differentiation of transgenic from non-transgenic roots by simple GUS staining. This is a significant improvement in methodology as this protocol facilitates the separation of transgenic from non-transgenic tissues to avoid dilution effects.

Our transcriptome analyses identified an array of candidate transcriptional regulatory targets that are associated with *GmABAS1* expression (Supplementary Table S3). Eventually, *Glyma.01G060300* was demonstrated to be the transcriptional regulatory target of GmABAS1. The binding of GmABAS1 to the promoter of *Glyma.01G060300* was confirmed by EMSA (Fig. 6), and reciprocal expression patterns were also observed between *GmABAS1* and *Glyma.01G060300* under osmotic stress (Fig. 8). These findings suggest the negative regulation of *Glyma.01G060300* by *GmABAS1* under osmotic stress, which in turn regulates the sensitivity to ABA.

ABA is an important stress hormone in plants (Sah *et al.*, 2016). A number of ABA sensitivity-related proteins such as ABI1, ABI2, ABI3, ABI4, and ABI5 have been well characterized (Koornneef *et al.*, 1984; Mönke *et al.*, 2004; Ma *et al.*, 2009; Reeves *et al.*, 2011; Skubacz *et al.*, 2016). Downstream of the ABIs, the ABI5-binding proteins (AFPs) have been reported to be negative regulators of ABA signalling by targeting ABI5 for ubiquitin-mediated degradation (Lopez-Molina *et al.*, 2003). The expression of *AFP* genes in turn is regulated by ABI and ABF transcription factors (Lynch *et al.*, 2017). Here in soybean, we demonstrated that GmABAS1, a 1R-subtype MYB transcriptional repressor, is an additional component of the ABA sensitivity regulatory network besides the above-mentioned proteins.

The putative functions of *GmABAS1* in ABA sensitivity were demonstrated in seeds and guard cells. *Arabidopsis thaliana* expressing *GmABAS1* had smaller stomatal apertures under ABA treatments compared with the wild type and the counterpart expressing the truncated *GmABAS1Δ* (Fig. 9C). Together with EMSA and the expression studies, this result supports the notion that GmABAS1 enhances ABA sensitivity, possibly under osmotic stress.

In summary, we have identified a 1R-subtype MYB transcriptional repressor gene, *GmABAS1* from soybean, and *Glyma.01G060300*, an annotated *AFP3* homologue, as its transcriptional regulatory target. GmABAS1 possibly functions to enhance ABA sensitivity under osmotic stress to help the plant to adapt to the changing growing environment.

## Supplementary data

Supplementary data are available at *JXB* online.

Table S1. DNA oligos used in this study.

Table S2. Vectors used in this study.

Table S3. Genes selected for GO enrichment analysis and GmABAS1-binding motif search.

Fig. S1. Alignment of coding sequence (CDS) regions showing the differences between *GmABAS1* and *GmABAS1∆* among the soybean accessions.

Fig. S2. Gene Ontology (GO) enrichment analyses of selected genes from the transcriptome.

Fig. S3. Competitive EMSA demonstrating the binding between GmABAS1 and the promoter region of *Glyma.01G060300*.

Fig. S4. Relative expression levels of *AtAFP1* in *A. thaliana* leaves under ABA treatment.

eraa081_suppl_Supplementary_Figures_S1-S4

eraa081_suppl_Supplementary_Tables_S1-S3
