# Supplementary material for: ABAS1 from soybean is a 1R-subtype MYB transcriptional repressor that enhances ABA sensitivity
Source: J Exp Bot. 2020 Feb 15;71(10):2970–81. doi: 10.1093/jxb/eraa081 (PMC7260724; doi:10.1093/jxb/eraa081)
Supplement: eraa081_suppl_Supplementary_Figures_S1-S4 [file eraa081_suppl_supplementary_figures_s1-s4.pdf]

```
                1                                                          60
C01             ATGGCAATGGCGCCTTCAACTTCCAATAGTGGGTCTGGAAAGAAGGTTAGAAAGCCTTAT
C02             ATGGCAATG-------------------GTGGGTCTGGAAAGAAGGTTAGAAAGCCTTAT
C08             ATGGCAATG-------------------GTGGGTCTGGAAAGAAGGTTAGAAAGCCTTAT
C12             ATGGCAATGGCGCCTTCAACTTCCAATAGTGGGTCTGGAAAGAAGGTTAGAAAGCCTTAT
C14             ATGGCAATG-------------------GTGGGTCTGGAAAGAAGGTTAGAAAGCCTTAT
C16             ATGGCAATGGCGCCTTCAACTTCCAATAGTGGGTCTGGAAAGAAGGTTAGAAAGCCTTAT
C17             ATGGCAATGGCGCCTTCAACTTCCAATAGTGGGTCTGGAAAGAAGGTTAGAAAGCCTTAT
C19             ATGGCAATGGCGCCTTCAACTTCCAATAGTGGGTCTGGAAAGAAGGTTAGAAAGCCTTAT
C24             ATGGCAATGGCGCCTTCAACTTCCAATAGTGGGTCTGGAAAGAAGGTTAGAAAGCCTTAT
C27             ATGGCAATGGCGCCTTCAACTTCCAATAGTGGGTCTGGAAAGAAGGTTAGAAAGCCTTAT
C30             ATGGCAATGGCGCCTTCAACTTCCAATAGTGGGTCTGGAAAGAAGGTTAGAAAGCCTTAT
C33             ATGGCAATG-------------------GTGGGTCTGGAAAGAAGGTTAGAAAGCCTTAT
C34             ATGGCAATGGCGCCTTCAACTTCCAATAGTGGGTCTGGAAAGAAGGTTAGAAAGCCTTAT
C35             ATGGCAATGGCGCCTTCAACTTCCAATAGTGGGTCTGGAAAGAAGGTTAGAAAGCCTTAT
W01             ATGGCAATGGCGCCTTCAACTTCCAATAGTGGGTCTGGAAAGAAGGTTAGAAAGCCTTAT
W02             ATGGCAATGGCGCCTTCAACTTCCAATAGTGGGTCTGGAAAGAAGGTTAGAAAGCCTTAT
W03             ATGGCAATGGCGCCTTCAACTTCCAATAGTGGGTCTGGAAAGAAGGTTAGAAAGCCTTAT
W04             ATGGCAATGGCGCCTTCAACTTCCAATAGTGGGTCTGGAAAGAAGGTTAGAAAGCCTTAT
W05             ATGGCAATGGCGCCTTCAACTTCCAATAGTGGGTCTGGAAAGAAGGTTAGAAAGCCTTAT
W06             ATGGCAATGGCGCCTTCAACTTCCAATAGTGGGTCTGGAAAGAAGGTTAGAAAGCCTTAT
W07             ATGGCAATGGCGCCTTCAACTTCCAATAGTGGGTCTGGAAAGAAGGTTAGAAAGCCTTAT
W08             ATGGCAATGGCGCCTTCAACTTCCAATAGTGGGTCTGGAAAGAAGGTTAGAAAGCCTTAT
W09             ATGGCAATGGCGCCTTCAACTTCCAATAGTGGGTCTGGAAAGAAGGTTAGAAAGCCTTAT
W10             ATGGCAATGGCGCCTTCAACTTCCAATAGTGGGTCTGGAAAGAAGGTTAGAAAGCCTTAT
W11             ATGGCAATGGCGCCTTCAACTTCCAATAGTGGGTCTGGAAAGAAGGTTAGAAAGCCTTAT
W12             ATGGCAATGGCGCCTTCAACTTCCAATAGTGGGTCTGGAAAGAAGGTTAGAAAGCCTTAT
W13             ATGGCAATGGCGCCTTCAACTTCCAATAGTGGGTCTGGAAAGAAGGTTAGAAAGCCTTAT
W14             ATGGCAATGGCGCCTTCAACTTCCAATAGTGGGTCTGGAAAGAAGGTTAGAAAGCCTTAT
W15             ATGGCAATGGCGCCTTCAACTTCCAATAGTGGGTCTGGAAAGAAGGTTAGAAAGCCTTAT
W16             ATGGCAATGGCGCCTTCAACTTCCAATAGTGGGTCTGGAAAGAAGGTTAGAAAGCCTTAT
W17             ATGGCAATGGCGCCTTCAACTTCCAATAGTGGGTCTGGAAAGAAGGTTAGAAAGCCTTAT
                *********                   ********************************
```

Fig. S1. Alignment of coding sequence (CDS) regions showing the differences between *GmABAS1* and *GmABAS1*Δ among the soybean accessions. C02, C08, C14 and C33 contain *GmABAS1*Δ while the other accessions contain *GmABAS1*.

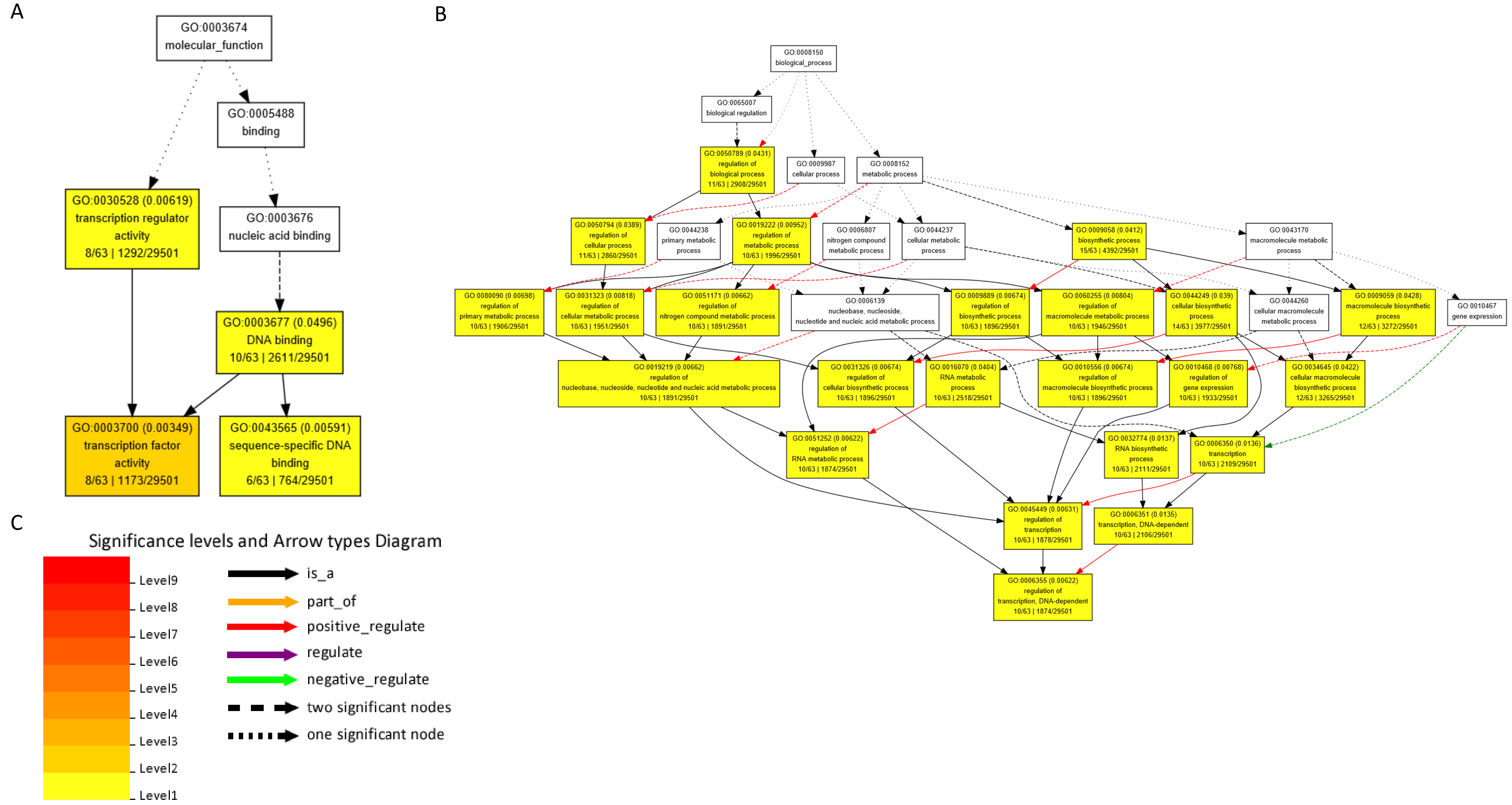

Fig. S2. Gene ontology (GO) enrichment analyses of selected genes from the transcriptome. (A) GO enrichment analysis in terms of molecular functions. (B) GO enrichment analysis in terms of biological processes. Genes having a lower expression level under ABA treatment when *cMyc-GmABAS1* was expressed compared to the empty vector control were subjected to GO enrichment analysis. (C) The keys of significance levels of GO enrichment and arrow types. Level 1-9 represent increasing significance levels. Each box represents a detected GO term. The *p*-value of each GO term is stated in brackets behind each GO ID. White boxes represent insignificant GO terms. The relationships among each GO term are denoted by different arrow types. At the bottom of each box of enriched GO term, the ratio at the left-hand side: the number of genes mapped to the GO term/the number of genes in the submitted query list; the ratio at the right-hand side: the background ratio of genes belonging to the GO term/total number of genes.

A

Zea mays ABI4-binding domain 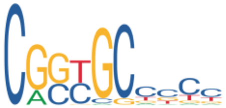

Glyma.01G060300 native probe -661 TGAAAAATCGTGTCAGTGTCGCCACGTG -634

Glyma.01G060300 mutated probe TGAAAAATCGTGTAAAAAAAAAAACGTG

B

-	108	-	-	-	-	-	-	-	GST (pmole)
-	-	72	72	72	72	72	72	72	GST-GmABAS1 (pmole)
10	10	10	10	10	10	10	10	10	6-FAM- native probe (pmole)
-	-	-	500	400	300	200	100	-	Unlabelled native probe (pmole)
-	-	-	-	-	-	-	-	500	Unlabelled mutated probe (pmole)

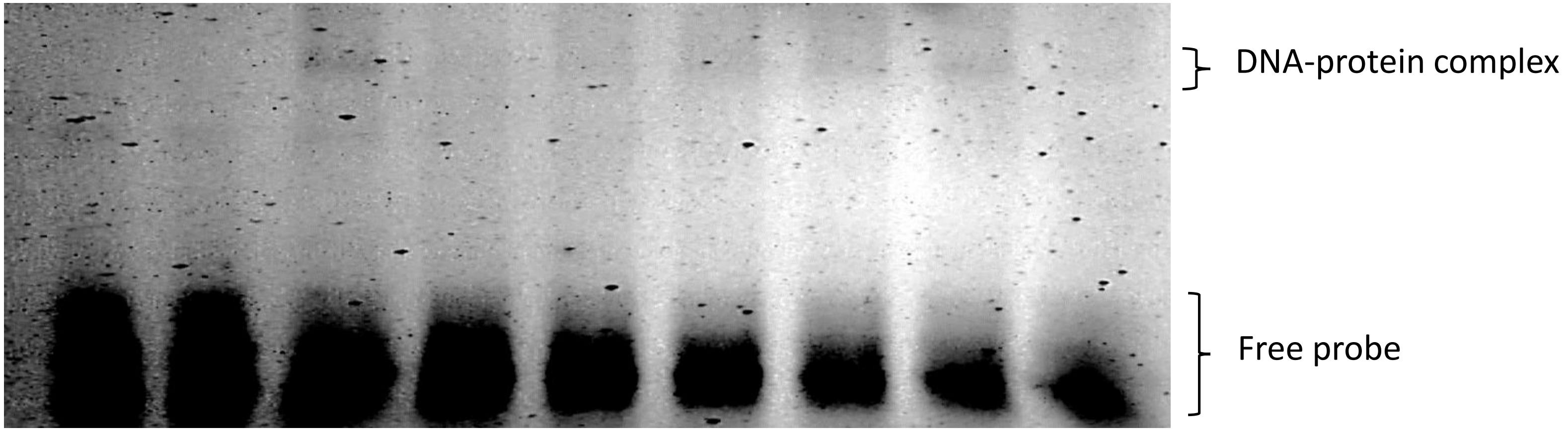

Fig. S3. Competitive electromobility shift assay (EMSA) demonstrating the binding between GmABAS1 and the promoter region of *Glyma.01G060300*. GmABAS1 was purified with a GST tag. Purified GST alone or GST-GmABAS1 was incubated with 6-FAM labelled native probes. Unlabelled native probes or unlabelled mutated probes were employed as the competitor. (A) Sequences of the native probe and the mutated probe. (B) The incubated protein-DNA probe mixture was electrophoresed in a 10% acrylamide gel. After that, the gel was visualized under UV using Gel Doc™ EZ Gel Imager (Bio-Rad). Retardation of the DNA probe migration was due to binding with GmABAS1.

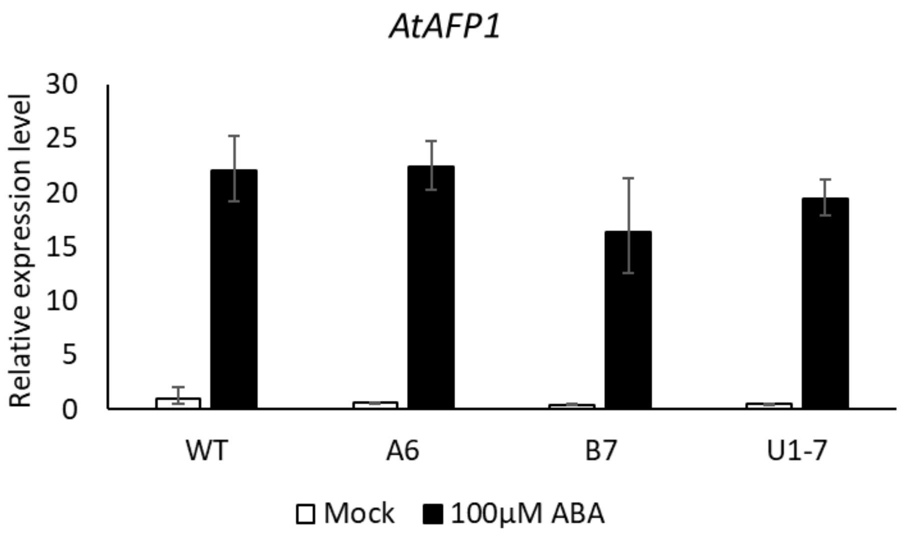

Fig. S4. Relative expression levels of *AtAFP1* in *A. thaliana* leaves under ABA treatment. Detached leaves of *A. thaliana* were treated with 100µM ABA or without ABA (mock) in the perfusion solution. The expression level of *AtAFP1* (Lynch *et al.*, 2016) was determined using qRT-PCR and normalized to that of *AtTUBβ* (At5g62690). Error bar represents the standard error of 3 technical repeats. WT: wild type; A6, B7: transgenic plants ectopically expressing GmABAS1; U1-7: transgenic plants ectopically expressing GmABAS1Δ.
