# Supplementary material for: ABAS1 from soybean is a 1R-subtype MYB transcriptional repressor that enhances ABA sensitivity
Source: J Exp Bot. 2020 Feb 15;71(10):2970–81. doi: 10.1093/jxb/eraa081 (PMC7260724; doi:10.1093/jxb/eraa081)
Supplement: eraa081_suppl_Supplementary_Tables_S1-S3 [file eraa081_suppl_supplementary_tables_s1-s3.pdf]

Table S1. DNA oligos used in this study

DNA oligo	Sequence (5’-3’)
Cloning of *GmABAS1* into V7, forward primer	GGTCTAGAATGGCAATGGCGCCTTCAAC
Cloning of *GmABAS1* into V7, reverse primer	GGCTCGAGTCAAGCTGTTTGACATGATAGAT
Cloning of *GmABAS1*Δ into V7, forward primer	GGTCTAGAATGGCAATGGTGGGTCTGG
Cloning of *GmABAS1*Δ into V7, reverse primer	GGCTCGAGTCAAGCTGTTTGACATGATAGAT
Cloning of *GFP* into *V7-GmABAS1*, forward primer	GGGTCTAGAATGGTGAGCAAGGGCGAGG
Cloning of *GFP* into *V7-GmABAS1*, reverse primer	AGTTCTAGACTTGTACAGCTCGTCCATGCC
Cloning of *GFP* into *V7-GmABAS1*Δ, forward primer	GGGTCTAGAATGGTGAGCAAGGGCGAGG
Cloning of *GFP* into *V7-GmABAS1*Δ, reverse primer	AGTTCTAGACTTGTACAGCTCGTCCATGCC
Cloning of *GmABAS1* into pMD20, forward primer	GGCTCGAGATGGCAATGGCGCCTTCAAC
Cloning of *GmABAS1* into pMD20, reverse primer	AAAAGTCGACTCAAGCTGTTTGACATGATAG
Cloning of *GmABAS1*Δ into pMD20, forward primer	GGCTCGAGATGGCAATGGTGGGTCTGG
Cloning of *GmABAS1*Δ into pMD20, reverse primer	AAAAGTCGACTCAAGCTGTTTGACATGATAG
Cloning of *AtVIP1* into pGBKT7, forward primer	AAAAAGAATTCATGGAAGGAGGAGGAAGAG
Cloning of *AtVIP1* into pGBKT7, reverse primer	AAAAGGATCCTCAGCCTCTCTTGGTGAAATC
Cloning of *AtDET1* into pGBKT7, forward primer	AAAACATATGATGTTCACAAGCGGTAACG
Cloning of *AtDET1* into pGBKT7, reverse primer	AAAAGGATCCTCATCGCCTAAAATGGATATTG
Cloning of *GmABAS1* into pLexA, forward primer	CACCATGGCAATGGCGCCTTCAA
Cloning of *GmABAS1* into pLexA, reverse primer	AAAAGTCGACTCAAGCTGTTTGACATGATAG
Cloning of *GmABAS1*Δ into pLexA, forward primer	CACCATGGCAATGGTGGGTCTGG

Cloning of *GmABAS1*∆ into pLexA, reverse primer	AAAAGTCGACTCAAGCTGTTTGACATGATAG
Cloning of *cMyc*-*GmABAS1* into pCambia3301, forward primer	GGCTCGAGTCACAGATCCTCTTCTGAGATGAGTTTTTGTTCAGCTGTTTGACATGATAGAT
Cloning of *cMyc*-*GmABAS1* into pCambia3301, reverse primer	AAAAGTCGACTCAAGCTGTTTGACATGATAG
Cloning of *GmABAS1* into pGEX-4T-1, forward primer	TTCCCGGGGATGGCAATGGCGCCTTCAAC
Cloning of *GmABAS1* into pGEX-4T-1, reverse primer	GGCTCGAGTCAAGCTGTTTGACATGATAGAT
Cloning of the promoter (3kb upstream to the translational start) of *Glyma.01G060300* into pLacZi, forward primer	GAAAGGAAATTCATATTTTTTTAAAAATGAAATCCATAAAAAGGTTATATTGTTAGGTTTAAAGCATAC
Cloning of the promoter (3kb upstream to the translational start) of *Glyma.01G060300* into pLacZi, reverse primer	CAATGCACGTTACCAAAGGCAAAACCACATAGAACATCGTGAAGTAGCAACACTTGCTGTTCTAATTCTAACG
Cloning of *cMyc*-*GmABAS1* into pBEVY-T, forward primer	GGCTCGAGTCACAGATCCTCTTCTGAGATGAGTTTTTGTTCAGCTGTTTGACATGATAGAT
Cloning of *cMyc*-*GmABAS1∆* into pBEVY-T, forward primer	GGCTCGAGGAACAAAAACTCATCTCAGAAGAGGATCTGATGGCAATGGTGGGTCTGG
Cloning of *cMyc*-*GmABAS1* or *cMyc*-*GmABAS1∆* into pBEVY-T, reverse primer	AAAAGTCGACTCAAGCTGTTTGACATGATAG
Amplification of *GmABAS1* or *GmABAS1*∆ from soybean germplasms, forward primer	GCTAACGGCTTGATAATGGGTG
Amplification of *GmABAS1* or *GmABAS1*∆ from soybean germplasms, reverse primer	TGCTAATGCTGCTGGTTGTGC
Detection of *GmABAS1* by ddPCR, forward primer	GACTTCTGATCTGAATAACTGTT
Detection of *GmABAS1* by ddPCR, reverse primer	CGCCTTCAACTTCCAATAGTG
Detection of *GmELF-1b* by ddPCR, forward primer	CCACTGCTGAAGAAGATGATGATG
Detection of *GmELF-1b* by ddPCR, reverse primer	AAGGACAGAAGACTTGCCACTC
Detection of *Glyma.01G060300* by ddPCR, forward primer	GCTGCCCACAGAGACCGA
Detection of *Glyma.01G060300* by ddPCR, reverse primer	GCTGCCCACAGAGACCGA

Detection of *Glyma.18G267200* by ddPCR, forward primer	GAAGGGAGCAATCCGGTGGA
Detection of *Glyma.18G267200* by ddPCR, reverse primer	CCACAAACACGTGCGGTCAA
Detection of *60s ribosomal protein* by ddPCR, forward primer	AAAGTGGACCAAGGCATATCGTCG
Detection of *60s ribosomal protein* by ddPCR, reverse primer	TCAGGACATTCTCCGCAAGATTCC
Detection of *AtAFP1* by qRT-PCR/ ddPCR, forward primer	GCTGGTCTCGATTCACTACAA
Detection of *AtAFP1* by qRT-PCR/ ddPCR, reverse primer	CTTCGTCACTGGACGCTTTA
Detection of *AtAFP2* by qRT-PCR/ ddPCR, forward primer	GAGGTAGTTCATCGAGCTTGTC
Detection of *AtAFP2* by qRT-PCR/ ddPCR, reverse primer	TCACACTCGGTCTCTCTGTT
Detection of *AtTUBβ* (At5g62690) by qRT-PCR/ ddPCR, forward primer	GCCAATCCGGTGCTGGTAACA
Detection of *AtTUBβ* (At5g62690) by qRT-PCR/ ddPCR, reverse primer	CATACCAGATCCAGTTCCTCCTCCC
Probe for EMSA—*Glyma.01G060300* native probe, sense strand	TGAAAAATCGTGTCAGTGTCGCCACGTG
Probe for EMSA—*Glyma.01G060300* native probe, antisense strand	CACGTGGCGACACTGACACGATTTTTCA
Probe for EMSA—*Glyma.01G060300* mutated probe, sense strand	TGAAAAATCGTGTAAAAAAAAAAACGTG
Probe for EMSA—*Glyma.01G060300* mutated probe, antisense strand	CACGTTTTTTTTTTTACACGATTTTTCA
Probe for EMSA— Oligo-T for oligo-T-oligo-A probe	TTTTTTTTTTTTTTTTTTTTTTTTTTTT
Probe for EMSA— Oligo-A for oligo-T-oligo-A probe	AAAAAAAAAAAAAAAAAAAAAAAAAAAA

Table S2. Vectors used in this study

Vector	Purpose
pMD20	Sub-cloning of genes into pGBKT7
pGBKT7	Expression of *GmABAS1* or *GmABAS1*Δ in the yeast strain Y2HGold
V7	Expression of *GFP*, *GFP-GmABAS1* or *GFP-GmABAS1*Δ
pLexA	Expression of *LexA-GmABAS1* or *LexA-GmABAS1*Δ in yeast strain W303a
JK1621	Reporter plasmid for LexA-based yeast transcriptional assay
pLGΔ312S	Negative control plasmid for LexA-based yeast transcriptional assay
pCambia3301	Expression of *cMyc-GmABAS1* in soybean hairy roots
pGEX-4T-1	Expression of *GST* or *GST-GmABAS1* in *E. coli* strain Rosetta2
pLacZi	Reporter plasmid for yeast promoter-binding assay
pBEVY-T	Effector plasmid for yeast promoter-binding assay

Table S3. Genes selected for GO enrichment analysis and GmABAS1 binding motif search

Differentially expressed genes identified from transcriptome analysis	Functional annotation	Fold of induction by ABA treatment in empty vector control (A)+	Fold of induction by ABA treatment in *cMyc-GmABAS1* expressers (B)+	Relative fold of repression due to *cMyc-GmABAS1* (A/B)
Glyma.07G149600	Cation exchanger 3	57.00	5.63	10.12
Glyma.17G013900	Protein of unknown function, DUF584	16.50	3.00	5.50
Glyma.18G025200	Pollen Ole e 1 allergen and extensin family protein	189.35	35.37	5.35
Glyma.09G231500	Glycosyl hydrolases family 32 protein	152.18	30.16	5.05
Glyma.13G282100	MYB-like 102	181.53	38.79	4.68
Glyma.20G041900	Ribonuclease 3	20.94	5.26	3.98
Glyma.08G170900	None	34.86	9.04	3.86
Glyma.12G204100	None	7.14	1.89	3.79
Glyma.04G010900	S-adenosyl-L-methionine-dependent methyltransferases superfamily protein	338.05	89.64	3.77
Glyma.11G120500	Protein of unknown function (DUF677)	10.44	2.79	3.75
Glyma.10G193400	Integrase-type DNA-binding superfamily protein	14.18	4.08	3.48
Glyma.04G246900	None	12.06	3.69	3.27
Glyma.13G138300	Peroxidase superfamily protein	6.74	2.14	3.15

Glyma.04G209200	Amino acid permease 2	27.50	8.89	3.09
Glyma.08G182700	Nodulin MtN21 /EamA-like transporter family protein	18.07	5.90	3.06
Glyma.20G011000	2-Oxoglutarate (2OG) and Fe(II)-dependent oxygenase superfamily protein	22.35	7.38	3.03
Glyma.06G125800	Nodulin MtN3 family protein	4.35	1.45	3.01
Glyma.03G159600	PRA1 (Prenylated rab acceptor) family protein	5.65	1.90	2.97
Glyma.19G141100	Remorin family protein	47.94	16.66	2.88
Glyma.12G027200	Glycosyl hydrolase 9C1	313.30	113.94	2.75
Glyma.07G179900	2-oxoglutarate (2OG) and Fe(II)-dependent oxygenase superfamily protein	15.41	5.69	2.71
Glyma.19G194000	Purple acid phosphatases superfamily protein	51.52	19.22	2.68
Glyma.19G069200	Highly ABA-induced PP2C gene 2	148.56	55.80	2.66
Glyma.03G251400	Phosphoenolpyruvate carboxylase kinase 2	25.46	9.61	2.65
Glyma.20G151700	Hydroxyproline-rich glycoprotein family protein	13.04	4.95	2.63
Glyma.03G116700	Integrase-type DNA-binding superfamily protein	10.58	4.05	2.61

Glyma.14G199900	None	45.22	17.38	2.60
Glyma.02G235100	SOS3-interacting protein 4	5.87	2.26	2.59
Glyma.13G243200	NAC domain containing protein 25	18.55	7.20	2.58
Glyma.09G012700	None	48.53	19.09	2.54
Glyma.11G012800	Protein of unknown function (DUF604)	20.02	7.92	2.53
Glyma.01G060300	ABI5 binding protein 3	57.28	22.68	2.53
Glyma.02G216200	None	96.34	39.25	2.45
Glyma.08G093600	Purple acid phosphatase 3	14.73	6.07	2.43
Glyma.03G186900	UDP-glucosyl transferase 73C2	260.86	108.02	2.41
Glyma.07G086300	Heat shock factor 1	38.24	16.18	2.36
Glyma.14G011800	RING/U-box superfamily protein	28.67	12.36	2.32
Glyma.08G178100	Aldolase-type TIM barrel family protein	68.00	30.03	2.26
Glyma.02G034200	ARM repeat superfamily protein	9.13	4.07	2.25
Glyma.17G048100	Inter-alpha-trypsin inhibitor heavy chain-related	5.51	2.49	2.22
Glyma.02G302400	RING/U-box superfamily protein	23.32	11.14	2.09
Glyma.07G257700	Aluminium induced protein with YGL and LRDR motifs	4.25	2.06	2.07
Glyma.03G255000	G-box binding factor 3	7.53	3.78	1.99
Glyma.09G190600	Heat shock transcription factor C1	25.17	12.71	1.98
Glyma.06G113100	None	5.08	2.60	1.95

Glyma.09G132200	Beta-hydroxylase 1	14.42	7.50	1.92
Glyma.08G181100	Xylem NAC domain 1	70.16	36.98	1.90
Glyma.14G189100	Concanavalin A-like lectin protein kinase family protein	8.02	4.24	1.89
Glyma.01G157700	Auxin efflux carrier family protein	8.99	4.75	1.89
Glyma.09G201200	Cinnamyl alcohol dehydrogenase 9	425.91	227.04	1.88
Glyma.03G168000	Pleiotropic drug resistance 12	181.66	97.09	1.87
Glyma.03G168000	Pleiotropic drug resistance 12	181.66	97.09	1.87
Glyma.04G152800	None	19.02	10.26	1.85
Glyma.10G010100	NIM1-interacting 1	7.44	4.03	1.84
Glyma.14G080500	Serine carboxypeptidase-like 40	24.09	13.15	1.83
Glyma.11G104200	Homolog of X-ray repair cross complementing 2 (XRCC2)	6.87	3.91	1.76
Glyma.18G072800	BTB/POZ domain-containing protein	10.49	6.01	1.75
Glyma.09G168300	Beta-amylase 1	12.61	7.30	1.73
Glyma.12G067800	Proline-rich family protein	9.34	5.41	1.73
Glyma.13G184500	Bromodomain 4	17.68	10.30	1.72
Glyma.07G191700	Protein of unknown function (DUF707)	6.99	4.12	1.70
Glyma.16G021000	Homeobox 7	275.07	163.29	1.68
Glyma.07G052100	Homeobox 7	97.98	58.68	1.67
Glyma.10G223500	Cellulose synthase 6	59.56	35.85	1.66

Glyma.04G079200	Hemoglobin 3	13.42	8.19	1.64
Glyma.18G267200	ABI5 binding protein 3	9.46	5.81	1.63
Glyma.17G061900	Homogentisate phytyltransferase 1	6.22	3.94	1.58
Glyma.06G040100	Polyketide cyclase / dehydrase and lipid transport protein	5.01	3.26	1.54
Glyma.16G179100	Beta-hydroxylase 1	115.95	77.03	1.51
Glyma.08G023100	UDP-D-glucose/UDP-D-galactose 4-epimerase 2	5.76	3.92	1.47
Glyma.06G310700	nodulin MtN21 /EamA-like transporter family protein	12.33	8.40	1.47
Glyma.20G091300	Low temperature and salt responsive protein family	23.43	16.50	1.42
Glyma.05G227100	Protein phosphatase 2C family protein	38.86	27.46	1.42
Glyma.07G058200	SPA (suppressor of phyA-105) protein family	3.52	2.52	1.40
Glyma.06G021200	DC1 domain-containing protein	102.90	74.19	1.39
Glyma.15G157700	S-adenosyl-L-methionine-dependent methyltransferases superfamily protein	14.25	10.57	1.35
Glyma.11G123000	Transducin/WD40 repeat-like superfamily protein	62.67	49.36	1.27

Glyma.10G218400	FORMS APLOID AND BINUCLEATE CELLS 1A	11.12	8.83	1.26
Glyma.14G066400	Protein phosphatase 2CA	13.01	10.85	1.20
Glyma.10G244000	Heat shock transcription factor A6B	4.46	3.95	1.13
Glyma.09G235500	None	9.64	10.43	0.92
Glyma.06G183800	ARM repeat superfamily protein	68.71	75.18	0.91
Glyma.10G228900	Protein kinase superfamily protein	110.39	136.08	0.81
Glyma.06G204100	ABI5 binding protein 3	79.66	128.01	0.62
Glyma.16G032200	ACC synthase 1	187.65	460.84	0.41
Glyma.18G273300	Myb domain protein 121	*	*	*
Glyma.08G156600	None	644.06	*	*
Glyma.02G147900	Aluminium activated malate transporter family protein	319.85	*	*
Glyma.13G365800	Cystathionine beta-synthase (CBS) protein	*	*	*
Glyma.05G126800	None	*	148.72	*
Glyma.11G232100	Pollen Ole e 1 allergen and extensin family protein	58.21	*	*

$^{+}$Average of two biological repeats

*The FPKM value of this gene was 0 without ABA treatment.
